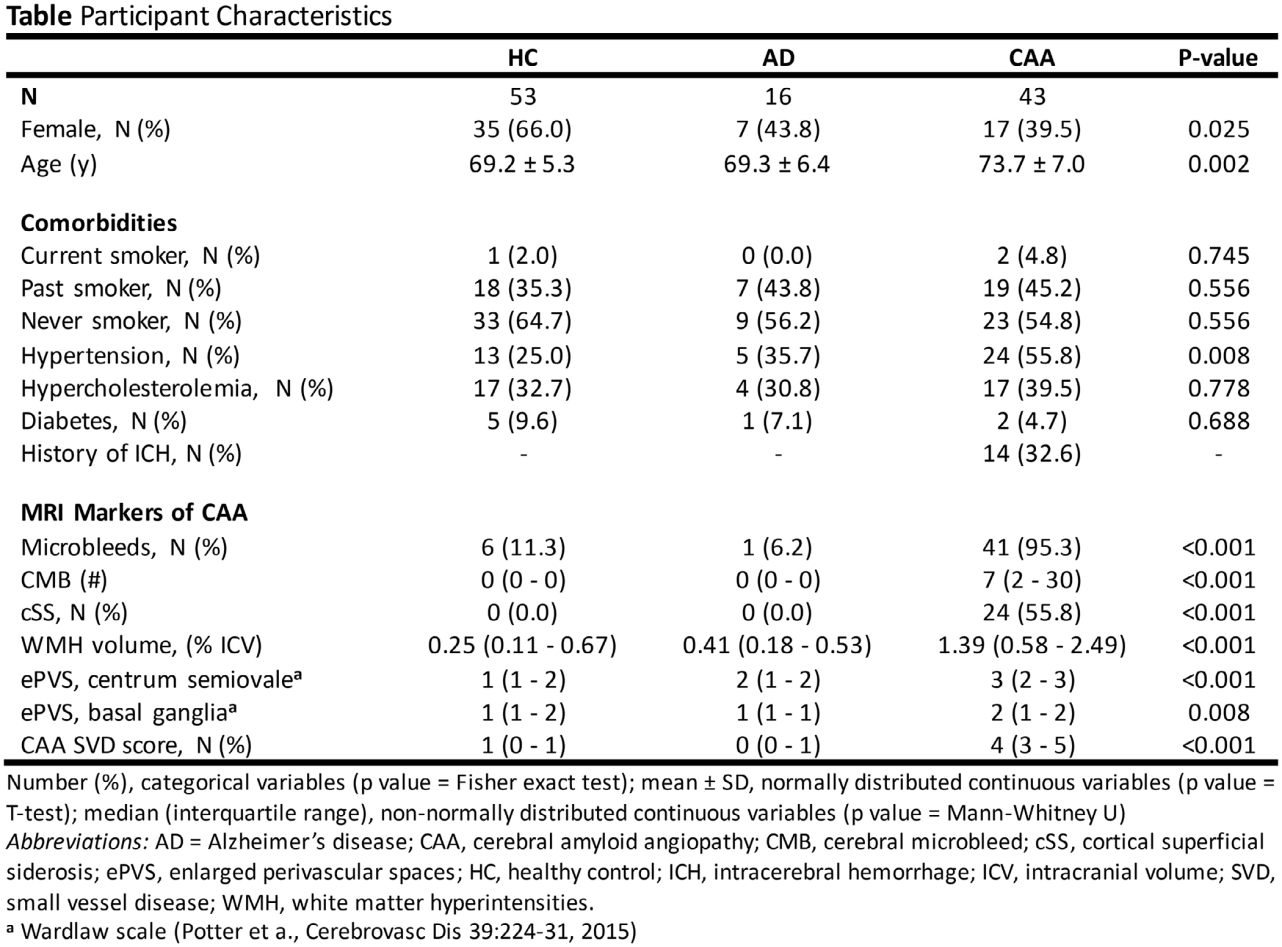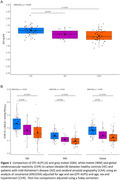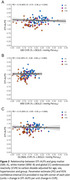# Association between glymphatic function and cerebrovascular reactivity in patients with cerebral amyloid angiopathy and Alzheimer's disease

**DOI:** 10.1002/alz70855_099499

**Published:** 2025-12-23

**Authors:** S Dahyun Park, Cheryl R. McCreary, Myrlene Gee, Erin L. Mazerolle, Richard Frayne, Zahinoor Ismail, Glen Jickling, Richard Camicioli, G. Bruce Pike, Eric E. Smith, Andrew E. Beaudin

**Affiliations:** ^1^ University of Calgary, Calgary, AB, Canada; ^2^ University of Alberta, Edmonton, AB, Canada; ^3^ St. Francis Xavier University, Antigonish, NS, Canada; ^4^ Hotchkiss Brain Institute, University of Calgary, Calgary, AB, Canada

## Abstract

**Background:**

Cerebral amyloid angiopathy (CAA) is characterized by deposition of amyloid‐beta (Aβ) within the walls of the small blood vessels of the brain and leptomeninges. It causes ∼20% of intracerebral hemorrhage, increases the risk of dementia, and is common in patients with Alzheimer's disease (AD). Deposition of Aβ may result from impaired glymphatic clearance concurrent with a reduction in cerebrovascular reactivity, but this has not been investigated in patients with CAA or AD.

**Method:**

Patients with CAA (*N* = 43) and AD (*N* = 16) patients and healthy controls (HC; *N* = 53) underwent an MRI at 3T that included a T1‐weighted and fluid attenuated inversion recovery (FLAIR) images, diffusion‐weighted imaging (30 directions; b‐value=1000s/mm^2^) and BOLD imaging involving a 2‐minute hypercapnic challenge (5% inspired CO_2_). Glymphatic function was quantified by diffusion tensor imaging along the perivascular space (DTI‐ALPS; ratio between diffusion in the perivascular space direction and diffusion of free water in the interstitium) while CVR was quantified as the % change in BOLD per mmHg increase in the end‐tidal partial pressure of CO_2_. Group comparisons of DTI‐ALPS were adjusted for age and sex while comparisons of CVR were adjusted for age, sex and hypertension. Associations between DTI‐ALPS and CVR were adjusted for age, sex, hypertension, white matter hyperintensity volume and group.

**Result:**

CAA and AD participants were predominantly male whereas HCs were predominantly female (Table). CAA participants were older with a greater prevalence of hypertension. DTI‐ALPS was lower in patients with CAA and AD versus HCs (ANCOVA, *p* <0.001; Figure 1A). Similarly, grey matter (GM), white matter (WM), and global CVR (mean of GM and WM) were lower in CAA and AD compared to HCs (all ANCOVAs, *p* <0.001; Figure 1B). However, DTI‐ALPS was not associated with GM (‐0.18 (‐0.73 ‐ 0.36), *p* = 0.504), WM (0.02, ‐0.84 ‐ 0.89, *p* = 0.646) or global CVR (‐0.15, ‐0.85 ‐ 0.55, *p* = 0.94; Figure 2).

**Conclusion:**

Vascular contributions to reduced glymphatic function in CAA and AD may be more related to changes in vasomotion and pulsatility of blood flow through cerebral vessels than the ability of the cerebrovasculature to dilate.